# Accurate Ultrasonic Thickness Measurement for Arbitrary Time-Variant Thermal Profile

**DOI:** 10.3390/s24165304

**Published:** 2024-08-16

**Authors:** Rajendra P. Palanisamy, Do-Kyung Pyun, Alp T. Findikoglu

**Affiliations:** Materials Physics and Applications (MPA), Los Alamos National Laboratory, Los Alamos, NM 87545, USA; dpyun@lanl.gov (D.-K.P.); findik@lanl.gov (A.T.F.)

**Keywords:** ultrasonic thickness measurement, corrosion monitoring, temperature compensation, transient thermal condition, real-time measurement

## Abstract

Ultrasonic thickness measurement of mechanical structures is one of the most popular and commonly used nondestructive methods for various kinds of process control and corrosion monitoring. With ultrasonic propagation speed being temperature-dependent, the thickness measurement can be performed reliably only when the thermal profile is completely known. Most conventional techniques assume the temperature of the test structure is uniform and at room temperature across its thickness. Such assumptions may lead to large errors in the thickness measurement, especially when there are significant temperature variations across the thickness. State-of-the-art techniques use external temperature measurements or implement iterative methods to compensate for the unknown thermal profiles. However, such techniques produce unsatisfactory results when the heat distribution is complex or varies rapidly with time. In this work, we propose a two-sensors technique, using both compressive and shear excitations, with a non-iterative rapid data processing method for accurate thickness measurement under arbitrary time-variant thermal profile. The independent behavior of shear and compressive waves is used to formulate a real-time thickness estimation technique. The developed technique is experimentally validated on a steel plate with fixed acoustic sensors. Test results show that the error in thickness estimation can be reduced by up to 98% compared to conventional thickness gauging methods.

## 1. Introduction

Ultrasonic thickness measurement is a well-known structural health monitoring tool used to detect corrosion or wall loss in vessels and pipelines [1,2,3,4,5,6,7]. It is also used in monitoring the etch rate in various industrial processes [8,9,10]. Compared to conventional thickness gauging techniques, ultrasonic thickness measurement offers improved precision and convenience since it requires access to only one side of the inspected wall [11]. The measurement principle relies on precise timing of the excitation from the front wall and reception of the ultrasonic pulse from the back wall (also known as time-of-flight (TOF) measurement) [12,13,14,15,16]. Most conventional methods assume that the propagation speed of the ultrasonic wave is constant during the measurement. However, with ultrasonic speed being sensitively dependent on temperature, this assumption leads to large errors in applications where the test structures possess any appreciable thermal gradients, which may also be time varying under normal operation conditions [17,18,19,20,21]. Thus, to reduce the measurement uncertainty, operations have to be stopped, and it must be ensured that the test structure under inspection is in an isothermal condition. This leads to potential process interruption and production loss.

Measuring the thermal profile precisely in real time is known to be extremely difficult due to the lack of access to the inner wall, as well as the lack of information on all the contributing heat sources and sinks influencing the thermal profiles within the thickness [20,22]. In conventional techniques, temperature compensation is most commonly achieved by measuring external wall temperature and assuming the structure is in an isothermal condition. Although such compensation techniques reduce the error in thickness estimation, large and rapidly changing thermal gradients within the inspected region lead to correspondingly increased thickness measurement errors. Recent research work by Zhang et al. used both shear and compressive sensors along with a one-dimensional heat diffusion model to predict the temperature at the inner wall and the temperature distribution across the wall thickness [9,20]. This method assumes that the heating source is only from the inner wall; thus, it becomes ineffective when a more complex heating/cooling mechanism is involved, leading to complex temperature profiles. Further, the iterative prediction process implemented in this work requires large computational power, which may be impractical for portable measurement systems. 

In this study, we propose a two-sensor approach combined with a novel thickness estimation technique that can measure wall thickness with high accuracy and precision under virtually any arbitrary thermal profile within the thickness. Due to the difference in wave propagation mechanism of compressive and shear modes, their dependence on temperature is also different [9,20]. Taking advantage of this phenomenon, the effect of virtually any thermal profile can be effectively compensated with this method. The proposed method uses a non-iterative simple formulation that can enable real-time temperature compensation for thickness measurement using portable systems. The real-time aspect of the method also allows for rapid temperature compensation even when fast temperature changes and corresponding highly time-variant temperature profiles exist in the inspected structure. The developed technique is tested on a stainless steel plate. Test results indicate that the error in thickness measurement can be lowered by as much as 98% compared to conventional techniques with no temperature compensation, and by approximately 75% compared to techniques that measure outer wall temperature and assume a uniform temperature profile. Further, the proposed method also shows robust performance under rapidly changing thermal conditions. 

This paper is organized as follows: First, the calibration of a selected steel plate and a general theoretical formulation to measure plate thickness is presented in Section 2. Next, the proposed thickness estimation technique is experimentally tested in Section 3. Test results and overall performance of proposed technique are discussed in Section 4. Finally, key findings of this work are summarized, and concluding remarks are presented in Section 5.

## 2. Calibration and Theoretical Formulation

This section describes the experimental procedure and the methods used to calibrate the stainless steel plate. Using the calibration results, theoretical formulations are developed to measure plate thickness under arbitrary sub-surface thermal profile. 

### 2.1. Calibration

Calibration in this work refers to identifying the relationship between the temperature and the speed of shear and the compressive ultrasonic wave.

#### 2.1.1. Experimental Setup and Procedure

**Figure 1:**
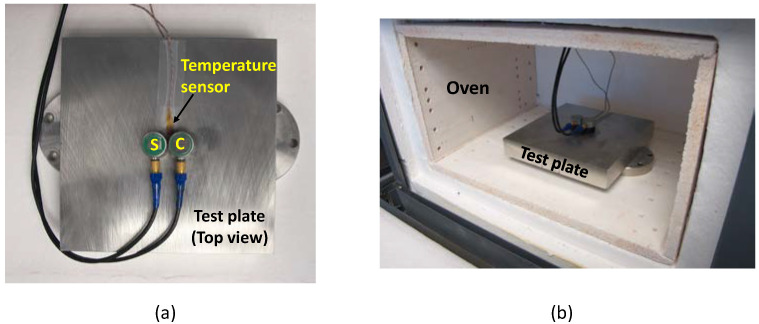
(**a**) Top view of the test plate with attached acoustic shear (S) and compressive (C) and thermocouple sensors. (**b**) Test plate along with attached sensors placed in the oven for calibration.

**Figure 2:**
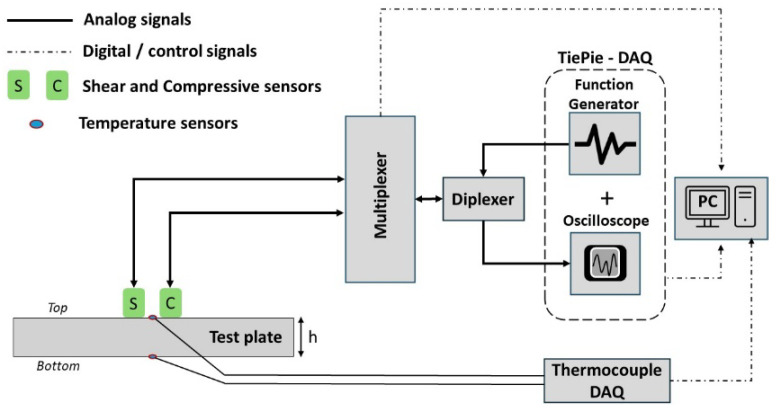
Schematic of the experimental setup to perform sequential pulse-echo measurement using shear (S) and compressive (C) acoustic sensors.

The plate chosen for this study is made of 304 stainless steel with a dimension of 15.24 × 15.24 cm and a thickness of 2.54 cm, as shown in Figure 1a. Compressive (Model no: V109) and shear (Model no: V153) acoustic sensors with central resonant frequency of 5 MHz from Evident Scientific, Inc. (Waltham, MA, USA) are attached using super glue, as shown in Figure 1a. Both sensors are operated under pulse-echo mode in a sequential manner to avoid interference. K-type thermocouple temperature sensors from Olympus Inc. (Waltham, MA, USA) are attached to the top and bottom of the plate, as shown in Figure 2. The experimental setup consists of four major components: (1) A Tie-Pie (model: HS5-540XMS-W5, Koperslagersstraat, Sneek, The Netherlands) data acquisition (DAQ) unit, equipped with an arbitrary waveform generator (AWG) and an oscilloscope, is used for the generation and acquisition of analog acoustic signals. A diplexer (model: RDX-6) from RITEC, Inc. (Warwick, RI, USA), connected to the DAQ, is used to isolate the echo signal during pulse-echo operation. (2) A multiplexer (model: 34980A) from Keysight (Santa Rosa, CA, USA) is used to switch the operation between compressive and shear sensors. (3) A Thermocouple DAQ is used to convert the analog thermocouple sensor data to digital data. (4) A personal computer (PC) with Python interface is used to control the DAQs, multiplexer and save the data.

**Figure 3:**
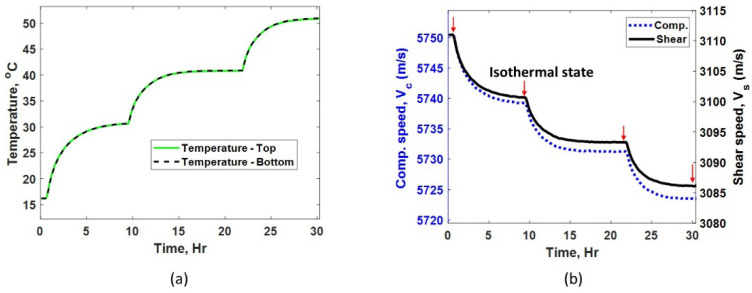
(**a**) Temperature measured at the top and bottom of the test plate during calibration. (**b**) Calculated compressive and shear speed in the test plate during calibration.

During calibration, the test plate is placed in an oven, as shown in Figure 1b. The temperature of the oven is increased from room temperature (18 °C) to 30, 40, and 50 °C, with at least 8 h between each increase to achieve steady state for high precision measurements. Temperature measured by thermocouples attached to the bottom and top of the plate are shown in Figure 3a. It can be observed that the plate surfaces reach steady state temperatures at 8 h. However, this does not ensure an isothermal condition through the plate thickness. In fact, ultrasonic waves propagating through the thickness of the plate can be used to sensitively determine any sub-surface thermal gradients. Also, as an additional check on the steady state condition, propagation speed of shear and compressive waves are tracked until they reach steady state, as shown in Figure 3b. For calibration purposes, we will assume that an isothermal condition has been achieved when the temperature and speed measurements have reached steady state and the difference between the top and bottom temperature readings has approached zero.

A Gaussian pulse with a center frequency of 5 MHz with 0.5 bandwidth (fractional bandwidth in the frequency domain of pulse) is used for the pulse echo measurements. Gaussian pulse generated by AWG is used to excite the acoustic sensor via diplexer and multiplexer. The multiplexer helps to switch the operation between shear and compressive modes electronically. The diplexer is used to isolate the echo signal during the pulse-echo operation, as shown in Figure 2. Acquired signals are sampled at 200 mega samples per second by the digitizing oscilloscope. Signals from thermocouples attached to the top and bottom of the test plate are digitized using a thermocouple DAQ (Model: USB-TC, from Digilent, Pullman, WA, USA). A PC with PYTHON software (Version: 3.11.7) is used to control all the equipment and store the data as follows. The temperature measurement of the plate and the pulse echo measurement using compressive and shear sensors are conducted in rapid succession in less than 8 s, allowing for near real-time capture of transient effects when non-steady-state temperature effects are studied, as discussed in the following sections. Finally, all the measured temperature and acoustic signals are stored in the PC. This procedure is repeated every 5 min during the calibration process. 

#### 2.1.2. Calibration Results

**Figure 4:**
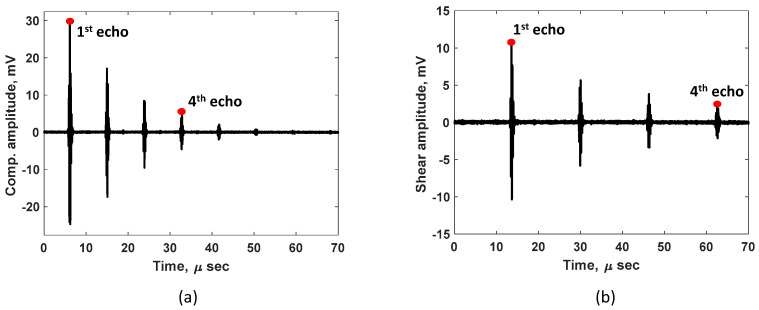
Acquired signal during pulse-echo measurement with (**a**) compressive and (**b**) shear acoustic sensors. The first and fourth echo used for TOF calculation are highlighted.

**Figure 5:**
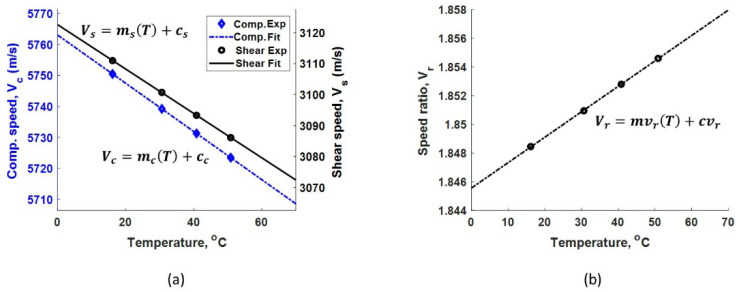
(**a**) Measured linear relationship between the temperature and the speed of shear and compressive modes. (**b**) Calculated linear relationship between the temperature and the speed ratio.

Figure 4a,b show the raw echo signals received using compressive and shear acoustic sensors, respectively. It can be observed that the shear pulse travels slower compared to the compressive pulse. This indicates the shear mode can be more sensitive for TOF measurements compared to the compressive mode. Due to having a limited sampling rate (200 M samples/s) and the use of a Gaussian pulse shape with the associated relatively large pulse width, it is beneficial to pick the farthest available echoes to help reduce the uncertainty (potential error) in TOF measurement, that is, until the signal-to-noise ratio becomes the limiting factor. Based on the echo signal amplitudes shown in Figure 4a,b, the first and the fourth echo of each mode were used to achieve the best measurement accuracy in our measurement set-up. The surface roughness of the test sample is expected to mainly affect the signal-to-noise ratio but may also lead to a significant spread of the time of arrival of the echo signal under certain conditions. A smooth surface is preferred to achieve the most reliable and accurate thickness estimation. The surface finish of the tested sample is 63 RMS (root mean square in microinches), which is considered an acoustically smooth surface finish since it is several orders of magnitude smaller than the acoustic wavelengths used. For a given plate thickness, which is separately measured using a micrometer, the calculated speed of compressive ($V_{c}$) and shear ($V_{s}$) modes are 5750 m/s and 3111 m/s, respectively, at the laboratory temperature of 16.2 °C. The speed is measured every 5 min during the calibration process, and the results are plotted in Figure 3b. A significant difference in the temperature dependence of compressive and shear speeds can be observed in Figure 5a (both y axes have same scale but different offsets); this indicates the different behavior of shear and compressive modes with temperature. Speed values measured only at the isothermal state are used for final calibration procedure. Figure 5a shows the linear fit for temperature-speed relationship. The linear fit for compressive mode has a slope ($m_{c}$) and incidence ($C_{c}$) of −0.77861 and 5763, respectively. Similarly, the linear fit for shear mode has a slope ($m_{s}$) and incidence ($C_{s}$) of −0.71681 and 3122.6, respectively, as shown in Figure 5a. It can be observed that the negative slope of both modes indicates the ultrasonic waves travel slower as the temperature of the plate increases [15]. However, the temperature–speed slopes are different for each ultrasonic mode. This precisely measured difference in temperature dependence is used to frame our unique thickness estimation technique. The speed ratio ($V_{r}$) calculated according to Equation (1) for compressive and shear modes with respect to temperature is shown in Figure 5b. The linear fit for speed ratio $V_{r}$ has a slope (${mv}_{r}$) and incidence (${cv}_{r}$) of 0.00017702 and 1.8456, respectively. It is important to note that this calibration automatically includes the temperature effects on both wave speed and physical thermal expansion.

(1) $$V_{r}=\frac{V_{c}}{V_{s}}$$

### 2.2. Theoretical Formulation for Thickness Estimation

**Figure 6:**
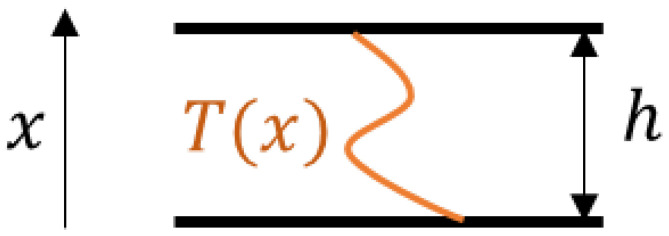
Schematic of test plate with arbitrary thermal profile T(x).

Ultrasonic speed in general is calculated, as shown in Equation (2), where *h* is the thickness of the test plate (see Figure 6). In the presence of sub-surface thermal gradient, the temperature of the plate in the thickness direction can be represented as T(x), where x ranges from 0 to h [20]. Equation (2) can be modified as Equations (3) and (4) for compressive and shear modes, respectively, where $V_{avg,c}$ and $V_{avg,s}$ are the average speed of compressive and shear modes, respectively. ${TOF}_{c}$ and ${TOF}_{s}$ are the time of flight for compressive and shear ultrasonic modes, respectively. The speed of the ultrasonic waves in Equations (3) and (4) are referred to as average speeds because a non-uniform thermal profile leads to non-uniform compressive and shear speeds within the plate thickness.

(2) $$V=\frac{2\times h}{TOF}$$

(3) $$V_{avg,c}=\frac{2\times h}{{TOF}_{c}}$$

(4) $$V_{avg,s}=\frac{2\times h}{{TOF}_{s}}$$

The ${TOF}_{c}$ and ${TOF}_{s}$ are obtained experimentally using compressional and shear acoustic sensors. However, the equation systems (3) and (4) cannot be solved as they have three unknown variables, i.e., two velocities ($V_{avg,c}$ and $V_{avg,s}$) and a plate thickness h. Equation (1), from calibration, establishes an empirical relationship between two speeds ($V_{avg,c}$ and $V_{avg,s}$), which provides the third equation to solve the equation system. The speed ratio $\left(V_{r}\right)$, calculated during calibration, was conducted at an assumed isothermal condition; thus, it is important to verify whether this empirical relationship is valid under any arbitrary thermal gradient T(x), as described in Equation (5).

(5) $$V_{r}=\frac{V_{c}}{V_{s}}=\frac{V_{avg,c}}{V_{avg,s}}=\frac{{TOF}_{s}}{{TOF}_{c}}$$

The average temperature of the plate in the thickness direction at a single location is given by $T_{avg}$, as shown in Equation (6).

(6) $$T_{avg}=\frac{1}{h}\int_{0}^{h}T\left(x\right)dx$$

Average speed of compressive ultrasonic mode can be written as shown in Equations (7)–(9) using Equation (6) and the linear fit obtained from calibration.

(7) $$V_{avg,c}=\frac{1}{h}\int_{0}^{h}\left[m_{c}\left(T\left(x\right)\right)+c_{c}\right]dx$$

(8) $$V_{avg,c}=m_{c}\frac{1}{h}\int_{0}^{h}\left(T\left(x\right)\right)dx+c_{c}$$

(9) $$V_{avg,c}=m_{c}T_{avg}+c_{c}$$

Similarly, average speed of shear mode is shown in Equation (10).

(10) $$V_{avg,s}=m_{s}T_{avg}+c_{s}$$

It can be observed from Equations (9) and (10) that the average temperature ($T_{avg}$) experienced by both the shear and the compressive waves are the same, and both modes have linear temperature–speed dependence. Therefore, we conclude that by solving the equation system 3–5, we can determine the plate thickness independent of any arbitrary subsurface thermal profile. It is important to note that the thermal profile T(x) is assumed to be the same for both shear and compressive waves during theoretical formulation. Thus, both sensors need to be kept close to each other to avoid any significant temperature variation under the sensors in the experimental setup (see Figure 1a). 

## 3. Experiments

**Figure 7:**
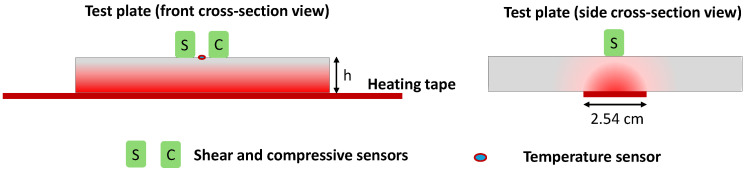
Schematic of experimental test setup (front and side cross-section view) that illustrates the placement of heating tape, acoustic sensor, and thermocouple. Red shading is used to indicate the existence of temperature gradients during validation experiments.

The thickness estimation technique developed in Section 2 is validated experimentally in Section 3, and the results are shown in Section 4. Figure 7 shows the schematic of the experimental setup. The data acquisition setup and calibration procedure were discussed previously, as shown in Figure 2. In the validation experiments, a heating tape with 2.54 cm width is placed in contact with the bottom of the test plate, right below the acoustic sensors. Temperature from the heating tape creates a subsurface thermal gradient, as shown in Figure 7. All the other edges and surfaces of the plate are exposed to room temperature. Unlike the procedure followed during the calibration, here, there is no equilibration time between measurements; instead, measurements are taken continuously while time-variant temperature profiles are introduced in the plate. During the validation tests, the thermocouple attached to the bottom of the plate is not used as it is also exposed to the heating tape, and measurements from the bottom plate may not be reliable. Also, it is important to note that the heating tape may not necessarily provide uniform temperature along the length of the tape. 

The thickness of the plate is calculated in real time using three different techniques. The first technique is the conventional method where the test plate is assumed to be at room temperature and no temperature compensation is performed; this method is referred to as the ‘no compensation’ method. In the ‘no compensation’ method, Equation (2), with room temperature wave speed of any one mode, is used to calculate the plate thickness. The second method uses one acoustic sensor, and the temperature is measured from the top of the plate to estimate plate thickness. In this method, the plate is assumed to be in an isothermal condition; this method is referred to as the ‘1-Sensor + temp’ method. In the ‘1-Sensor + temp’ method, Equation (2), with speed corresponding to measured temperature from the top surface, is used to calculate the plate thickness with rudimentary temperature compensation. Due to the high sensitivity of shear sensors, as discussed in Section 2, the above two methods use the shear mode for thickness estimation. Finally, the proposed ‘2-sensor’ method uses the following four steps to calculate the thickness with improved temperature compensation, notably without the need to measure temperature directly.

Step (1)—calculate the experimental speed ratio ($V_{r}$) using the measured TOF from shear and compressive sensors, as shown in Equation (5).
Step (2)—using the temperature and speed ratio relationship from calibration, calculate the average temperature ($T_{avg}$) in the plate.
Step (3)—use $T_{avg}$ in Equations (9) or (10) to calculate the average speed of compressive or shear ultrasonic waves, respectively. To ensure an unbiased comparison with other two methods, shear speed is also used here in the following step.
Step (4)—obtain the thickness of the plate from the calculated shear speed.

## 4. Results and Discussions

This section compares the measured plate thickness using the three thickness measurement methods discussed above, which are (1) no compensation, (2) 1-sensor + temp, and (3) 2-sensor. Two different heating mechanisms are tested in this work. The first method is continuous heating, where the heating tape is turned on for about 40 min continuously. A steadily equilibrating thermal gradient is expected to develop in this case. The second method is intermittent heating, where the heating tape is turned on and off repeatedly to introduce a more complex and rapidly changing thermal profile. 

### 4.1. Continuous Heating

**Figure 8:**
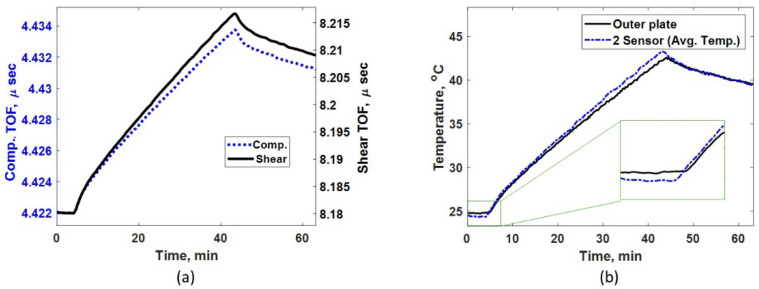
(**a**) Measured TOF for compressive and shear sensors under continuous heating. (**b**) Comparing measured temperature using thermocouple attached to the outer plate and indirect average temperature predicted via the two-sensor method under continuous heating.

**Figure 9:**
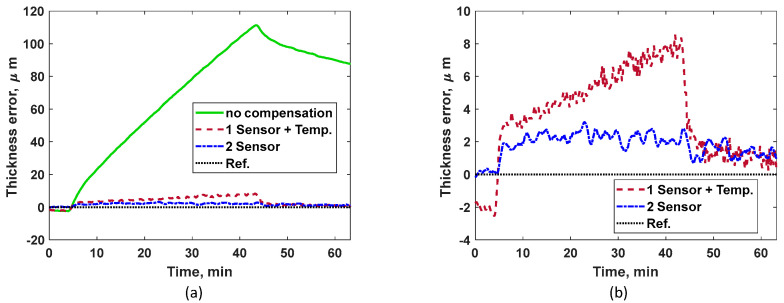
(**a**) Thickness measurement error for all three methods shown under continuous heating. (**b**) Measurement errors for the ‘1-sensor + temp’ method and the proposed ‘2-sensor’ methods, separately compared for better visualization.

Figure 8a shows the measured TOF using compressive and shear sensors for continuous heating case. A steady increase in TOF for both ultrasonic modes are noticed until the heat is turned off at approximately 43 min, which indicates that the ultrasonic speed reduces as the plate becomes hotter. Figure 8b shows the measured temperature at the outer plate and the average temperature predicted by the two-sensor technique using Step (2). Throughout the heating phase (between 4 and 43 min), the average temperature is higher than the outer plate temperature; this is because the plate never attains isothermal condition. However, as the plate cools down (after the heat is turned off at 43 min), the outer plate temperature approaches the average plate temperature, indicating the plate is approaching isothermal condition. Figure 8b includes a zoom in for initial temperature estimation. It can be observed that the initial average temperature of the plate is slightly lower than the plate’s outer surface temperature. This is because the plate surface is gradually heating up first as the room temperature increases. Even such minor effects that could lead to measurement errors can be successfully compensated by the two-sensor method, as shown in Figure 9. Also, when the heating begins, the outer plate temperature measurement could not recognize the initiation of the heating as thermal wave takes time to reach the other side of the plate. However, the two-sensor technique instantly identifies the heating process when it begins on the other side of the plate. 

Thickness measurement for the two-sensor method is calculated as described in Step (4). The error in thickness is the difference between predicted and actual plate thicknesses (*h*). The thickness error for all three methods considered is shown in Figure 9a. The error for the non-compensated method is about 111 µm, which is much larger those in the other two techniques. For better visualization, Figure 9b compares the ‘1-sensor + temp’ with our proposed ‘2-sensor’ technique. Based on the results in Figure 9b, the overall performance of the two-sensor method is significantly superior to that of the ‘1-sensor + temp’ method. A consistent accumulation of measurement error (maximum error of about 8 µm) is noticed with the ‘1-sensor + temp’ method due to the increase in the build-up of sub-surface thermal gradient as the heating progresses. As expected, the two-sensor method being immune to sub-surface thermal gradients shows a much smaller and constant error of about 2 µm in thickness measurement. Overall, in these validation tests, the error in thickness measurement for the two-sensor technique is 98% smaller than that of the ‘no compensation’ technique and 75% lower than that of the ‘1 sensor + temp’ technique. The measurement error noticed using the two-sensor method is possibly due to the non-uniform temperature of the contact areas along the heating tape, which might lead to slightly different surface temperatures and thermal gradients under the shear and compressive sensors. The initial error of about 2 µm in the ‘1-sensor + temp’ method, as shown in Figure 9b, is likely due to the gradual heating of the plate surface as the room temperature increases. However, results from the two-sensor method in Figure 9b clearly demonstrate that such environmental effects have been compensated successfully. 

### 4.2. Intermittent Heating

**Figure 10:**
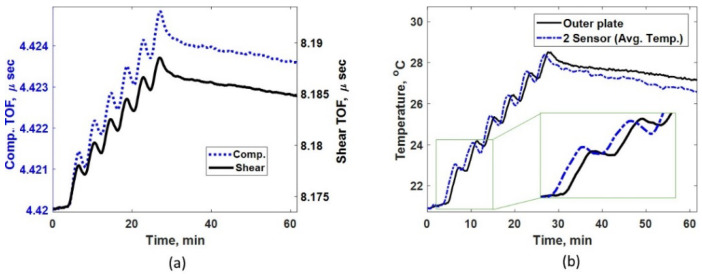
(**a**) Measured TOF for compressive and shear sensors under intermittent heating. (**b**) Comparing measured temperature using thermocouple attached to the outer plate and the indirect average temperature predicted by the two-sensor method under intermittent heating.

Figure 10a shows the measured TOF using compressive and shear sensors for the intermittent heating case. Figure 10b shows the measured temperature at the outer plate and the average temperature predicted by the two-sensor technique. The average temperature leads the outer plate temperature in time, indicating that the two-sensor technique tracks the heating or cooling process in real time as it occurs on the other side of the plate. Figure 10b includes a zoom in for initial temperature estimation to illustrate, clearly, the performance of the two-sensor technique under rapid heating and cooling cycles. Unlike the continuous heating case, the outer plate temperature and average temperature do not converge to the same values instantly after final cooling phase. This effect is due to the presence of complex sub-surface thermal gradients due to intermittent heating and additional laboratory temperature drifts. 

**Figure 11:**
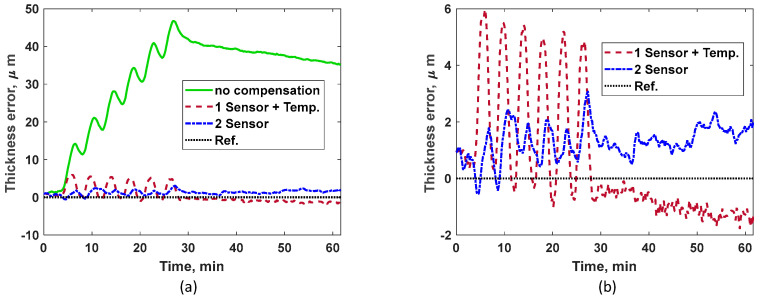
(**a**) Thickness measurement errors for all three methods shown under intermittent heating. (**b**) Measurement errors for the ‘1 sensor + temp’ method and the proposed ‘2-sensor’ method are separately compared for better visualization.

Thickness errors for all three methods considered are shown in Figure 11a. The error in the non-compensated method increases and fluctuates greatly as the plate undergoes intermittent heating, as shown in Figure 10b. For better visualization, Figure 11b compares the ‘1-sensor + temp’ and our proposed ‘2-sensor’ technique. Based on the results, the overall performance of the two-sensor method is superior, especially during intermittent heating, demonstrating the ability to handle rapid temperature fluctuations. Large variations in thickness measurements for the ‘1 sensor + temp’ method illustrate the weakness of conventional temperature compensation techniques. 

In this current demonstration, fixed sensors are used on a calibrated test plate. Future work will investigate the use of portable sensors and improvements on calibration, where speed changes due to temperature and material thermal expansion may need to be decoupled for improved accuracy. 

## 5. Summary and Conclusions

In this work, we have proposed an ultrasonic two-sensor thickness measurement technique. The different and near-linear dependency of shear and compressive ultrasonic propagation speeds on temperature allowed us to formulate a unique thickness measurement technique that automatically compensates for temporal and spatial variations in temperature in the inspected structure in real time. The effectiveness of the technique is verified by the initial calibration and validation tests. Theoretical formulations are developed to offer an accurate thickness measurement under an arbitrary sub-surface thermal profile, which could also be time-variant. Initial test results using permanently attached sensors indicate that the overall error in thickness measurement is reduced by 98% compared to the traditional measurement technique, with no temperature compensation, and by 75% compared to another measurement technique with a temperature compensation method using one surface temperature measurement. Further, the developed technique demonstrates a high tolerance for rapidly changing sub-surface thermal profiles. Due to the simple sensor deployment, rapid signal processing, and thickness measurement process, this method has the potential for widespread industrial application. 

## Data Availability

The original contributions presented in the study are included in the article, further inquiries can be directed to the corresponding author.
